# High sperm DNA stainability might not be an accurate predictive indicator of male fertility and assisted reproductive technology outcomes

**DOI:** 10.3389/fendo.2025.1510114

**Published:** 2025-02-26

**Authors:** Liyan Shen, Ce Zhang, Gaigai Wang, Xu Fu, Shenmin Yang, Jiaxiong Wang

**Affiliations:** Center for Reproduction and Genetics, The Affiliated Suzhou Hospital of Nanjing Medical University, Suzhou Municipal Hospital, Suzhou, China

**Keywords:** infertility, SCSA, DFI, HDS, ART outcomes

## Abstract

**Background:**

The clinical need for assisted reproduction continued to increase, so did the need for predictive markers of assisted reproductive technology (ART) outcomes. Among all the markers, sperm DNA integrity was paid more and more attention in the assessment of male fertility in recent years, but its clinical value remains still in doubt.

**Methods:**

We conducted a retrospective cohort study. Couples coming to our reproductive center were retrospectively enrolled, and semen and assisted reproductive technology parameters were assessed. Sperm DNA integrity was analyzed using a flow cytometric method. Statistics were analyzed to investigate the relationship of this on semen quality and ART outcomes.

**Results:**

DNA fragmentation index (DFI) was affected by abstinence days and age, and was a directly correlated with sperm quality parameters (p<0.001). Meanwhile high sperm DNA stainability (HDS) showed an unexplainable negative correlation with abstinence days (p<0.05), age (p<0.001) and body mass index (p<0.01).For sperm quality parameters, HDS showed a similar relevance besides abnormal sperm head morphology (p<0.001). Embryo cleavage and implantation rates were significantly negatively related to DFI in fresh intracytoplasmic sperm injection cycles (p<0.01) while HDS showed a positive relationship with high quality embryo rate (p<0.05). For final outcomes, only the live birth rate from fresh intracytoplasmic sperm injection cycles was positively correlated with DFI which is meaningless (p<0.05).

**Conclusions:**

HDS might not be an appropriate marker for male fertility and further studies are needed to identify the efficiency of SDF in clinical practice.

## Introduction

Male factors account for about 40-50% of infertility cases (1). The most common cause of male infertility is sperm abnormalities. Therefore, at present, a large part of laboratory tests for male fertility are focused on sperm quality analysis, such as sperm motility, morphology, viability. However, sperm quality is often not accurately reflected by a single test value (2). Sperm DNA fragmentation (SDF), as a complement to traditional sperm quality analysis, is being paid increasing attention in the clinic. Unlike those of somatic cells, sperm nucleoproteins undergo a histone-to-protamine transition during maturation, which causes a drastic change in the DNA topology, relieving torsional stress. In this progress, chromatin is remodeled by inducing double-strand breaks and their subsequent relegation. SDF increases significantly when this process is aberrant (3). There are various causes of increasing SDF, including apoptosis, defective maturation, oxidative stress, which are affected by many risk factors such as advanced paternal age, diet, life style, chemo-/radio- therapy, obesity, environmental toxicants, infection and testicular trauma (4).

There are many methods for the clinical detection of sperm SDF, including comet assay (5), sperm chromatin dispersion (SCD) (6), terminal deoxynucleotidyl transferase mediated dUTP nick end labeling (TUNEL) (7) and sperm chromatin structure assay (SCSA) (8, 9), the latter two methods being widely used in the clinic. The SCSA test is a high-precision test based on flow cytometry, which needs a large capital expense and a trained technician (10). In contrast to SCSA, the other methods are time-consuming and labor intensive (11). The values obtained by flow cytometry with the commercial SCSA test kit are the DNA fragmentation index (DFI) and high DNA stainability (HDS), which are considered to be indicators of the degree of sperm DNA integrity and sperm chromatin condensation, respectively. With SCSA, sperm DNA breaks can be evaluated indirectly through DNA denaturability. This assay is based on the characteristics of Acridine Orange (AO), which is a cell-permeable dye that shows fluoresces green when bound to native double-stranded DNA and yellow/red when bound to single-stranded DNA (12). Sperm samples were stained and analyzed by flow cytometry, producing a scatter plot, of the ratio of the number of green and red sperm heads. DFI is defined as the percentage of the red spermatozoa, while that of green spermatozoa was defined as HDS (13). Many studies have shown the correlation between traditional semen parameters values and, unexplained recurrent miscarriage, natural conception rates, assisted reproductive technology outcome and DFI (14–17). A few studies have supported the opposite view (18). Contrary to the fact that there is a large amount of data supporting the predicted value of DFI, the clinical definition and significance of HDS are ambiguous (19). In the early years, there were positive views on the predictive value of HDS (20), but recent reports have challenged it (21, 22). Although this parameter was debatable, there were few relevant large-scale statistical studies in recent years. Studies paid more attention on the indication of HDS on sperm quality, and the few studies focusing on assisted reproductive outcomes had a relatively low cycle count.

Here, we assessed the common factors affecting sperm DNA integrity, and how DFI and HDS reflected the sperm quality and ART outcome. It was found that HDS might not be an appropriate indicator of male fertility and assisted reproductive technology outcomes. Our findings provide an experimental basis and guidance for the clinical application of SDF indicators.

## Materials and methods

### Study design and participants

This is a single-center, retrospective, observational study. A total of 3970 couples were selected, who had undergone assisted reproductive treatment, including artificial insemination by husband (AIH), *in vitro* fertilization (IVF) and intracytoplasmic sperm injection (ICSI), in the reproductive center of our hospital from April 2018 to April 2021.

Inclusion criteria: (1) the ovulation induction program was standard or conventional long-term; (2) the age of the female patient was less than 35 years old; (3) both patients had no chromosomal abnormality, and the only factor was the Fallopian tube.

Exclusion criteria: (1) the female patient has reproductive system diseases; (2) the body mass index (BMI) of female patient was abnormal (greater than 24 or less than 18.5); (3) the woman had genetic defects, chronic diseases, etc. (4)the female patient has smoking habit; (5)the male patient has abnormal testicular size. This study was approved by the ethics committee and the patients gave informed consent for participation.

### General inspection of semen quality

Semen samples were collected by masturbation after 2–7 days of sexual abstinence and were processed for analysis after liquefaction for 60 min at 37°C. Sperm concentration, morphology and motility were assessed according to the WHO laboratory manual for the examination and processing of human semen (5th edition). Spermatozoa on slides were stained with Papanicolaou. 200 cells were morphologically assessed per smear.

### Sperm DNA fragmentation test

DFI was quantified by the SCSA kit (Zhejiang Cellpro Biotech, Ningbo, China). First, the liquefied semen was diluted with 4°C buffer to a sperm concentration of 1×10^6^/mL; secondly, 500 μL of acid solution was added to the diluted sperm suspension and after 30 seconds the dye acridine orange (AO) was added. The flow cytometer was calibrated, and then each sample was measured continuously at least twice in a carousel by the Navios flow cytometer (Beckman Coulter, USA), recording at least 5000 cells in each tube; the gate was set and the cytogram populations were determined according to the previous reference (10). Finally DFI and HDS were calculated with DFI View software for statistical analysis.

### ART procedures and outcomes

For AIH, the husband’s semen sample was obtained by masturbating for 2–7 days of sexual abstinence and optimized by density gradient centrifugation according to the protocol of the optimization kit (Irvine Scientific, Santa ANA, USA) with a final sperm concentration of 20 × 10^6^/mL. Then, the sperm suspension was injected into the female’s uterine cavity.

Conventional gonadotropin-releasing hormone agonists (GnRH-ant, Merck & Co., Ltd., USA), recombinant human follicle stimulating hormone (MerckSerono, USA) were used to induce ovulation. For IVF, the sperm concentration was adjusted to that in the AIH protocol, and added into the culture droplets. Meanwhile the oocytes were added to the prepared fertilization droplets 3-4 h after retrieval and fertilization was assessed at 16-18 h. For ICSI, after oocyte retrieval, the spermatozoa selected from the microscopic field were injected by using an ICSI platform. After fertilization, embryo morphology was scored according to the shape, size, spatial distribution of blastomeres, and cytoplasmic homogeneity.

In order to determine pregnancy, hCG in blood and urine was detected on the 14th day after embryo transfer; positive samples indicated a biochemical pregnancy, and clinical pregnancies were confirmed by B-ultrasound 4-6 weeks after embryo transfer. The outcome parameters of interest were rate of fertilization, embryo cleavage (number of fertilized cleavage embryos/number of fertilized ova)*100%), high-quality embryo (at day 3 of embryo culture, embryos with 6-12 cells and uniform cell size were categorized as good quality embryos), implantation, pregnancy and live births.

### Statistical analysis

SPSS 19.0 (IBM, USA) was used for data analysis. Measured data that were normally distributed were expressed as median (95% confidence interval). T-test was used to compare the differences in DFI and HDS between groups with different smoking habits (at least 3 cigarettes per day). Multivariate linear regression analysis was performed, using abstinence day, BMI and age as the independent variables and DFI, HDS as dependent variables to investigate the impact of these three factors on SDF. Linear regression and correlation analysis was performed, using DFI and HDS as the independent variable and semen parameters as dependent variables, to investigate the predictive value of SDF on sperm quality. The correlation of DFI, HDS and rates of IVF/ICSI fertilization, embryo cleavage, high-quality embryos and implantation were analyzed by the Spearman Rho test. The difference in implantation rates was analyzed by t-Test. The difference in pregnancy rate and live birth rate was analyzed by Chi-squared test; P<0.05 was considered statistically significant. Receiver operating characteristic (ROC) curves were constructed for analyzing the sensitivity and specificity of DFI and HDS for predicting seminal quality. The effect was evaluated from the area under the curve (AUC).

## Results

**Figure 1:**
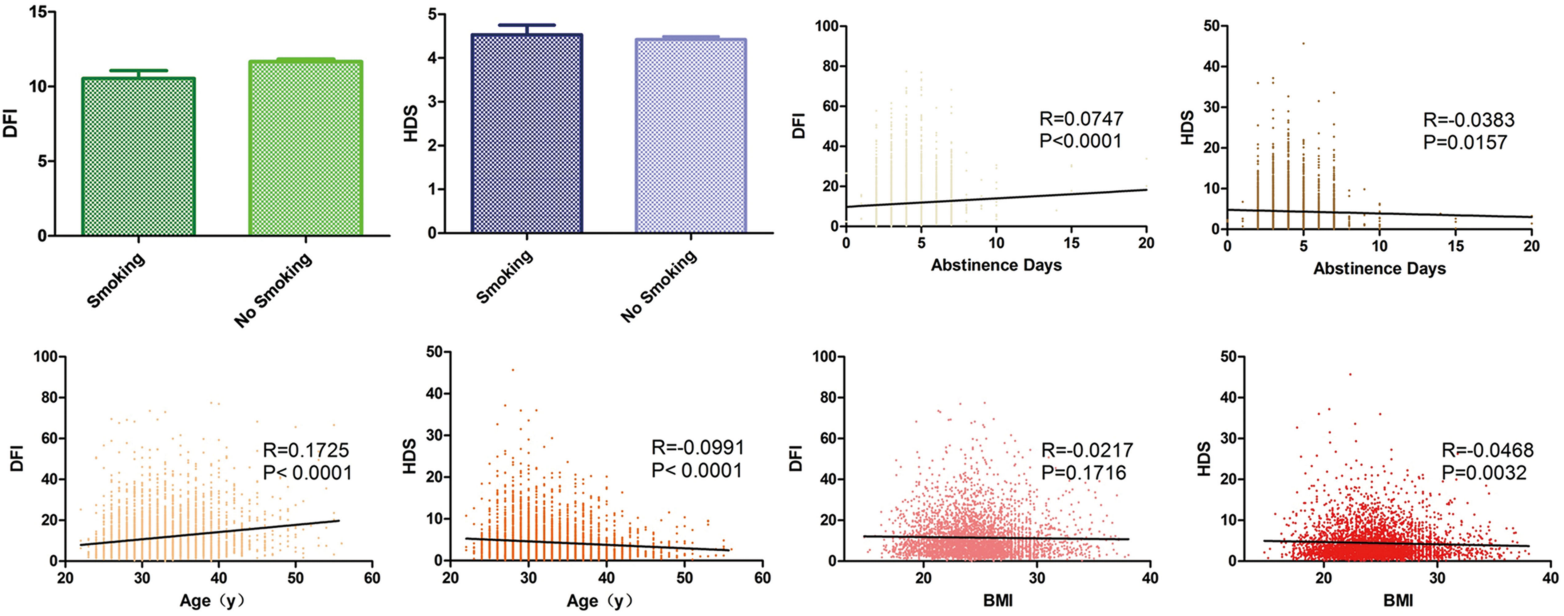
Analysis of SDF impact factors. Column grams representing the effect of smoking on DFI (green) and HDS (blue). Box plots showing correlation between DFI, HDS and abstinence days (yellow), age (orange) and BMI (red). The black line shows the correlation trend. R means correlation coefficient and P means p value.

11648 ART cycles were examined and the detailed information is shown in **Table 1**. The influence of factors on SDF revealed that DFI was increased by the increasing of abstinence and age (p < 0.001). HDS was decreased by increasing abstinence (p < 0.05), male age (p < 0.001) and BMI (p < 0.01). Smoking had no significant effect on either of these parameters (**Figure 1**).

**Table 1:** Couples characteristics and semen parameters classified according to the World Health Organization manual (5th).

Overall cohort	
Number of total cycles/couples	11648/3970
Number of cycles/couples included in the final cohort	6422 (2367)
Duration of infertility (years)	3 (1- 8)
Age	
Female age (years)	31 (25- 39)
Male age (years)	31 (26- 41)
Advanced maternal age, n. (%)	607 (15.3%)
BMI	
Female BMI (kg/m^2^)	21.97 (18.03- 28.55)
Male BMI (kg/m^2^)	24.33 (19.15- 30.78)
Smoking habit	
Female smoking habit, n, (%)	2 (0.1%)
Male smoking habit, n, (%)	317 (8.0%)
Anatomical anomalies	
Tubal problem, n, (%)	53 (1.4%)
Abnormal uterine morphology, n, (%)	86 (2.2%)
Abnormal testicular size, n, (%)	23 (0.6%)
Semen parameters	
Abstinence days	4 (2- 7)
Sperm concentration (mlion/mL)	43.6 (7.55- 16.71)
Total motility (%)	43.3 (14.1- 76.2)
Progressive sperm motility (%)	35.6 (11.0- 65.59)
Normal sperm morphology (%)	3 (0.5- 9.5)
Abnormal sperm head morphology (%)	92.5 (80- 99)
DFI	8.58 (2.26- 31.64)
HDS	3.14 (0.83- 12.50)

BMI, Body Mass Index; DFI, DNA Fragmentation Index; HDS, High DNA Stainability.

Data are reported as median (95% confidence interval).

**Figure 2:**
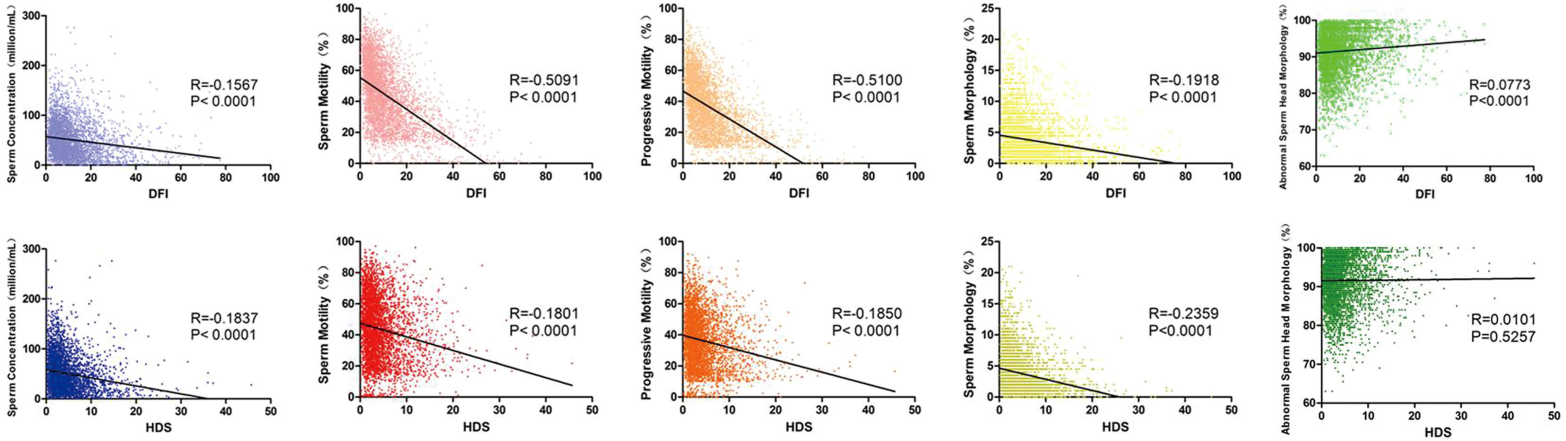
Linear regression and correlation analysis of SDF and sperm quality parameter values. Box plots showing correlation between DFI (upper panel), HDS (lower panel) and sperm concentration (blue), motility (red), progressive motility (orange), sperm morphology (yellow) and abnormal sperm head morphology (green). The black line shows the correlation trend. R means correlation coefficient and P means p value.

**Figure 3:**
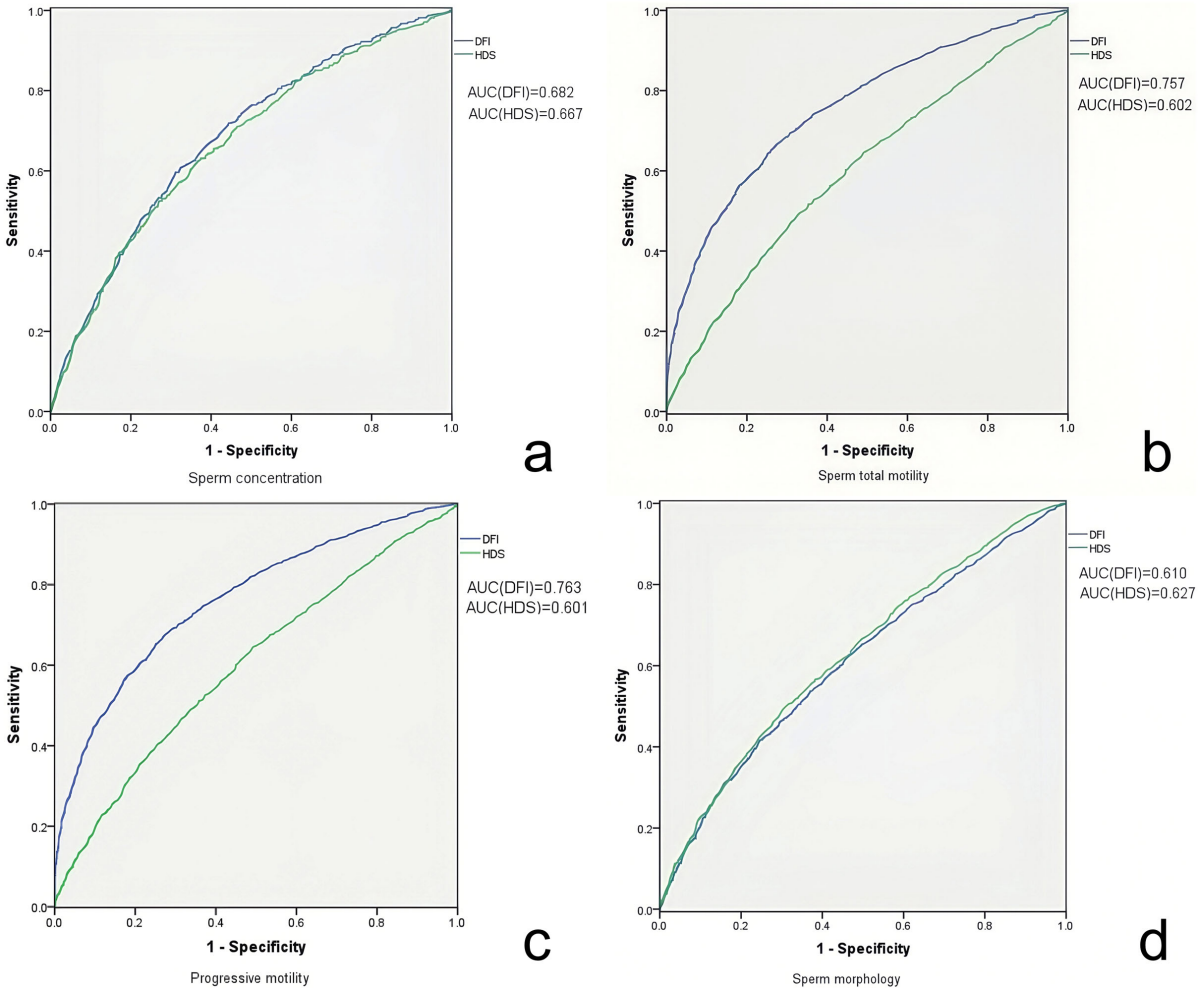
ROC curve analyzing the sensitivity and specificity of DFI and HDS for seminal quality. ROC curve of DFI (blue) and HDS (green) evaluating the sperm concentration **(a)**, total motility **(b)**, progressive motility **(c)** and morphology **(d)**. The areas under the curve of each ROC curve are indicated respectively in the figure.

To explore the predictive value of SDF for sperm quality, linear regression was used for visualizing correlation trends and a significant correlation between DFI and all the sperm quality parameters (p < 0.001) was found. As DFI increased, sperm concentration, motility, progressive motility and normal morphology decreased, whereas the abnormal sperm head percentage increased. HDS showed a similar correlation and trend with sperm concentration, motility, progressive motility and normal morphology (p < 0.001), but no correlation with the abnormal sperm head (**Figure 2**). From ROC curves of DFI and HDS on seminal quality, the AUC were from 0.6 to 0.8, it is worth noting that the AUC of DFI was larger than that of HDS for total and progressive sperm motility (**Figure 3**).

For the correlation between SDF and ART outcomes of 6422 cycles, the data are shown in **Table 2**. We used the Spearman Rho test to investigate the effect of DFI and HDS on ART parameters. In fresh IVF cycles, DFI was not directly related to rate of fertilization, cleavage, high quality embryos or implantation. There was a significant association of HDS with the high quality embryo rate. In ICSI fresh cycles, DFI was found negatively correlated with cleavage rate and implantation rate (p<0.01), while HDS showed no correlation (**Table 3**).

**Table 2:** Assisted reproductive technology (ART) outcomes.

	AIH cycles (n=922)	Fresh IVF cycles (n=1875)	Freeze-thaw IVF cycles (n=1785)	Fresh ICSI cycles (n=968)	Freeze-thaw ICSI cycle (n=872)
Oocytes inseminated (IVF fresh cycles) or injected (ICSI fresh cycles)	–	10 (2- 24)	–	7 (1- 20)-	–
Oocytes fertilized	–	6 (1- 16)	–	5 (0- 16)-	–
Total embryos	–	4 (0- 11)	–	3 (0- 11)	–
High quality embryos	–	3 (0- 11)	–	3 (0- 11)	–
Transferred embryos	–	1 (0- 2)	–	1 (0- 2)	–
Fertilization rate (%)	–	63.6 (21.9-100)	–	79.5 (0-100)	
Cleavage rate (%)	–	100 (80-100)	–	100 (0-100)	–
High quality embryos rate (%)	–	66.7 (0-100)	–	62.5 (0-100)	–
Number of cycles with transferable embryos	–	718	–	291	–
Biochemical pregnancy rate (%)	10.5 (97/922)	61.0 (438/718)	64.5 (1151/1785)	59.45 (173/291)	64.8 (565/872)
Clinical pregnancy rate (%)	8.8 (81/922)	52.4 (376/718)	54.9 (980/1785)	52.6 (153/291)	53.4 (465/872)
Live birth rate (%)	64.2 (52/81)	79.8 (300/376)	73.0 (715/980)	79.7 (122/153)	67.7 (315/465)

AIH, Artificial Insemination by Husband; IVF, *In Vitro* Fertilization; ICSI, Intracytoplasmic Sperm Injection.

Data are reported as median (95% confidence interval).

**Table 3:** Correlation analyses of SDF parameters and ART outcome parameters, sorted by *in vitro* fertilization (IVF), fresh intracytoplasmic sperm injection (ICSI) cycles.

Fresh IVF cycles				
	DFI		HDS	
	Correlation coefficient	*p*-value	Correlation coefficient	*p*-value
Fertilization rate	0.039	0.094	-0.033	0.156
Cleavage rate	0.019	0.406	-0.005	0.814
High quality embryo rate	0.018	0.439	0.047^*^	0.040
Implantation rate	-0.060	0.110	0.004	0.855
Fresh ICSI cycles				
	DFI		HDS	
	Correlation coefficient	*p*-value	Correlation coefficient	*p*-value
Fertilization rate	0.058	0.070	0.005	0.878
Cleavage rate	-0.086^**^	0.007	0.005	0.879
High quality embryo rate	-0.005	0.878	-0.039	0.221
Implantation rate	-0.179 ^**^	0.002	0.057	0.330

DFI, DNA Fragment Index; HDS, High DNA Stainability; IVF, *In Vitro* Fertilization; ICSI, Intracytoplasmic Sperm Injection.

**: *p<*0.01; *: *p*<0.05.

We divided the pregnancy and live birth data according to the DFI (DFI ≤ 15% for normal, 15%< DFI<30% for subnormal, DFI≥30% for abnormal) and HDS (HDS ≤ 15%, HDS>15% for abnormal). In the fresh ICSI group, the embryo implantation rate of patients with normal DFI was significantly higher than the subnormal DFI group (p<0.05) and abnormal normal (p<0.01). For the correlation between SDF and pregnancy rate, live birth rate, chi-square test was used, but only the live birth rate from fresh ICSI cycles showed a significant difference between those with normal and abnormal DFI (**Table 4**).

**Table 4:** T-test of implantation rate and Chi-squared analyses of clinical pregnancy rate, and live birth rate, sorted artificial insemination by husband (AIH), *in vitro* fertilization (IVF), Intracytoplasmic sperm injection (ICSI) cycles.

Fresh IVF cycles	Reference	Implantation rate (%)	*p* value		
DFI	a: ≤15%	13.4	a vs b		0.832
	b:>15% and <30%	12.9	b vs c		0.837
	c:≥30%	12.1	a vs c		0.886
HDS	≤15%	13.5			0.710
	>15%	15.1			
Fresh ICSI cycles	Reference	Implantation rate (%)	*p* value		
DFI	a: ≤15%	18.2	a vs b		0.018*
	b:>15% and <30%	11.9	b vs c		0.124
	c:≥30%	7.1	a vs c		0.004**
HDS	≤15%	9.7			0.640
	>15%	8.0			
Freeze-thaw IVF cycles	Reference	Implantation rate (%)	*p* value		
DFI	a: ≤15%	36.7	a vs b		0.355
	b:>15% and <30%	33.8	b vs c		0.166
	c:≥30%	24.5	a vs c		0.054
HDS	≤15%	36.1			0.396
	>15%	29.7			
Freeze-thaw ICSI cycles	Reference	Implantation rate (%)	*p* value		
DFI	a: ≤15%	31.0	a vs b		0.439
	b:>15% and <30%	33.7	b vs c		0.168
	c:≥30%	41.9	a vs c		0.054
HDS	≤15%	32.9			0.767
	>15%	31.2			
Freeze-thaw ICSI cycles	Reference	Implantation rate (%)	*p* value		
DFI	a: ≤15%	9.0 (68/752)	a vs b	0.227	0.634
	b:>15% and <30%	7.8 (11/141)	b vs c	0.028	0.867
	c:≥30%	6.9 (2/29)	a vs c	0.158	0.691
HDS	≤15%	8.0 (80/1002)		0.219	0.639
	>15%	12.5 (1/8)			
Fresh IVF cycles	Reference	Pregnancy rate (%)	χ value		*p* value
DFI	a: ≤15%	52.8 (319/604)	a vs b	0.113	0.736
	b:>15% and <30%	51.0 (51/100)	b vs c	0.328	0.568
	c:≥30%	42.9 (6/14)	a vs c	0.544	0.461
HDS	≤15%	52.8 (372/705)		2.476	0.110
	>15%	30.8 (4/13)			
Fresh ICSI cycles	Reference	Pregnancy rate (%)	χ value		*p* value
DFI	a: ≤15%	50.0 (81/162)	a vs b	0.029	0.886
	b:>15% and <30%	51.1 (46/90)	b vs c	2.670	0.102
	c:≥30%	66. 7 (26/39)	a vs c	3.507	0.061
HDS	≤15%	52.9 (145/274)		0.221	0.639
	>15%	47.1 (8/17)			
Freeze-thaw IVF cycles	Reference	Pregnancy rate (%)	χ value		*p* value
DFI	a: ≤15%	54.9 (822/1497)	a vs b	0.006	0.941
	b:>15% and <30%	54.7 (135/274)	b vs c	0.030	0.864
	c:≥30%	56.1 (23/41)	a vs c	0.023	0.880
HDS	≤15%	55.0 (962/1748)		0.597	0.440
	>15%	48.7 (18/37)			
Freeze-thaw ICSI cycles	Reference	Pregnancy rate (%)	χ value		*p* value
DFI	a: ≤15%	52.2 (277/531)	a vs b	0.035	0.851
	b:>15% and <30%	52.9 (138/261)	b vs c	2.294	0.130
	c:≥30%	62.5 (50/80)	a vs c	2.985	0.084
HDS	≤15%	53.5 (434/811)		0.168	0.684
	>15%	50.8 (31/61)			
AIH	Reference	Live birth rate (%)	χ value		*p* value
DFI	a: ≤15%	66.2 (45/68)	a vs b	3.573	0.059
	b:>15% and <30%	36.4 (4/11)	b vs c	2.758	0.097
	c:≥30%	100.0 (2/2)	a vs c	1.008	0.316
HDS	≤15%	62.5 (50/80)		0.596	0.440
	>15%	100.0 (1/1)			
Fresh IVF cycles	Reference	Live birth rate (%)	χ value		*p* value
DFI	a: ≤15%	79.9 (255/319)	a vs b	0.324	0.569
	b:>15% and <30%	76.5 (39/51)	b vs c	1.788	0.181
	c:≥30%	100.0 (6/6)	a vs c	1.499	0.221
HDS	≤15%	80.1 (298/372)		2.224	0.136
	>15%	50.0 (2/4)			
Fresh ICSI cycles	Reference	Live birth rate (%)	χ value		*p* value
DFI	a: ≤15%	72.8 (59/81)	a vs b	4.652	0.031*
	b:>15% and <30%	89.1 (41/46)	b vs c	0.310	0.578
	c:≥30%	84.6 (22/26)	a vs c	1.484	0.223
HDS	≤15%	80.0 (116/145)		0.117	0.732
	>15%	75.0 (6/8)			
Freeze-thaw IVF cycles	Reference	Live birth rate (%)	χ value		*p* value
DFI	a: ≤15%	73.8 (607/822)	a vs b	0.716	0.397
	b:>15% and <30%	70.4(95/135)	b vs c	1.742	0.187
	c:≥30%	56.5 (13/23)	a vs c	3.436	0.061
HDS	≤15%	73.1 (703/962)		0.368	0.544
	>15%	66.7 (12/18)			
Freeze-thaw ICSI cycles	Reference	Live birth rate (%)	χ value		*p* value
DFI	a: ≤15%	65.3 (181/277)	a vs b	0.740	0.390
	b:>15% and <30%	69.6 (96/138)	b vs c	0.742	0.389
	c:≥30%	76.0 (38/50)	a vs c	2.175	0.140
HDS	≤15%	67.7 (294/434)		0	1
	>15%	67.7 (21/31)			

DFI, DNA Fragment Index; HDS, High DNA Stainability; AIH, Artificial Insemination by Husband; IVF, *In Vitro* Fertilization; ICSI, Intracytoplasmic Sperm Injection.

**:*p<*0.01; *p<0.05.

## Discussion

As described above, various factors are related to SDF, such as life habit, BMI, male age and abstinence days. In this cohort, the smoking habit had no effect on DFI and HDS results contrary to a previous report (23). But apart from this, DFI was directly related to male age and abstinence days. The older the man was, the more the abstinence days, the higher the DFI. On the other hand, the opposite relationship of age, abstinence days and BMI with HDS shows a lack of clinical predictive value and support of evidence-based medicine for HDS.

DFI had been considered to be a reliable indicator reflecting sperm quality, on the basis of the strong connection with sperm motility (24), morphology (25) and ART outcomes (26). Nevertheless, there are a few reports supporting the opposite view (18). The results observed in our study showed a strong link between DFI and sperm parameter values. The sperm concentration, total and progressive motility, and normal sperm morphology were lower when DFI was higher, which could be explained by the sperm head’s composing mainly sperm nucleus, whose morphology would be affected by the organization of its DNA. DFI was not associated with IVF outcomes, consistent with several other studies, which make the predictive value of DFI an open question (27). The correlation between DFI and cleavage and implantation rates in fresh ICSI cycles indicates the predictive value of DFI on ICSI embryo quality, which has been mentioned in many studies (28). ART outcome of pregnancy and live births showed no relationship with DFI in our study; the only relationship found for DFI in fresh ICSI cycles was inexplicable. As shown in the retrospective cohort study of Deng et al., high DFI does not influence live birth, miscarriage or clinical pregnancy rates (15). There was also no significant difference in the pregnancy rate in intrauterine insemination cycles in another study (29). However, the reports of negative predictive results of DFI remain in the minority. It was confusing that our results showed a negative correlation of DFI with seminal quality but no clear association with reproductive results. We attribute these results to the fact that the DFI obtained here may not fully represent the DNA integrity of the spermatozoon that was ultimately used for ART, and the variability of sperm quality affected the reliability of the analysis. In addition, although we tried to eliminate female factors by inclusion and exclusion criteria, there might still be some shortcomings. Among the various studies on SDF, it is believed by some researchers that sperm DNA fragmentation on the day of fertilization is not associated with ART outcome independently of gamete quality (30). Well designed further studies are still needed.

HDS, as an indicator of the degree of chromatin maturation has been doubted recently. Although HDS values were simultaneously obtained and significantly correlated with the DFI values, before we doubted its clinical value, as HDS was thought to be independent from DFI. Recently, Lu published a short communication pointing out that HDS should not be recommended as a marker for the detection of sperm DNA damage. In his point of view, the establishment of HDS in the detection of sperm DNA damage has no theoretical basis and has also no support from evidence-based medicine (19). However, there were few studies to prove Lu’s point through clinical data statistics. As mentioned in our study, when used to reflect sperm quality, HDS performs well. But the loose association of HDS with normal sperm head morphology cannot be explained. Although several studies had affirmed the predictive value of HDS on ART outcomes, our result showed a poor relationship. Similarly, the positive relationship between HDS and IVF outcomes is confusing, implying the lower the chromatin maturity, the better the ART result. The definition of HDS is questionable; the strong green fluorescence may only represent the integrity of the sperm DNA, and not directly reflect the completion of sperm nucleoprotein replacement. HDS should be ranked by comparing it with other sperm nucleoprotein markers, like transition nuclear proteins 1 (TNP1), pre-protamine 2 (pPRM2), etc. Our statistics raise a question about the clinical use of HDS, and it is hoped that more data studies will emerge to demonstrate whether the clinical value of HDS is overestimated.

In summary, according to our results DFI is still an efficient marker of sperm DNA integrity, while HDS is not so useful. Although whether to choose HDS as a predictor in clinical laboratory is debatable, we must admit that the patients with high HDS had less chance to get their partner pregnant in most reports. Here, we present our data, but cannot refute HDS as an influence factor. We have to face the fact that there were limitations in our research. From the hundreds of men, very few had an HDS >15%, the low number made the statistical analysis results not convincing enough to deny the value of HDS. New markers are still needed to investigate the sperm DNA damage more precisely. Lately, the research team of Huazhong University of Science and Technology proposed a novel parameter, the mean number of sperm DNA breakpoints (MDB), which relies on a novel secondary amplification detection system they developed. The system is based on terminal deoxynucleotidyl transferase and endonuclease IV, which can effectively reflect the number of 3’-OH (equivalent to the number of breakpoints) (31). In Lu’s article, he improved the flow cytometry detection method of DFI through optimizing the conditions of gating (19). We also tried new flow cytometric parameters, such as the heterogeneity of seminal cell size distribution, which has been proved to be a good predictive effect on sperm quality. Nowadays, methods are emerging for assessment of sperm quality. It follows that many indicators have been to rapidly used in clinical testing without good evidence-based medical certification. SDF may reflect sperm quality, but still needs data validation, strict and scientific quality control and improvement of methods and parameters.

## Data Availability

The original contributions presented in the study are included in the article/supplementary material. Further inquiries can be directed to the corresponding author.
